# Coexisting Infinite Orbits in an Area-Preserving Lozi Map

**DOI:** 10.3390/e22101119

**Published:** 2020-10-03

**Authors:** Houzhen Li, Kexin Li, Mo Chen, Bocheng Bao

**Affiliations:** School of Microelectronics and Control Engineering, Changzhou University, Changzhou 213164, China; 19080902871@smail.cczu.edu.cn (H.L.); 16417103@smail.cczu.edu.cn (K.L.); mchen@cczu.edu.cn (M.C.)

**Keywords:** discrete maps, coexisting orbits, initial values, complexity, hardware platform

## Abstract

Extreme multistability with coexisting infinite orbits has been reported in many continuous memristor-based dynamical circuits and systems, but rarely in discrete dynamical systems. This paper reports the finding of initial values-related coexisting infinite orbits in an area-preserving Lozi map under specific parameter settings. We use the bifurcation diagram and phase orbit diagram to disclose the coexisting infinite orbits that include period, quasi-period and chaos with different types and topologies, and we employ the spectral entropy and sample entropy to depict the initial values-related complexity. Finally, a microprocessor-based hardware platform is developed to acquire four sets of four-channel voltage sequences by switching the initial values. The results show that the area-preserving Lozi map displays coexisting infinite orbits with complicated complexity distributions, which heavily rely on its initial values.

## 1. Introduction

Chaotic systems have long been a subject of concern in academic and industrial fields [1,2]. Due to the advantages of the ergodicity, unpredictability, pseudo-randomness and initial value sensitivity [3,4], chaotic systems fit well to various chaos-based image encryptions [5,6] and secure communications [7].

The coexistence of self-excited attractors or hidden attractors has been found in various kinds of continuous ordinary differential systems. These ordinary differential systems contain the purely mathematical chaotic and hyperchaotic systems [8,9], memristor-based chaotic circuits and systems [10,11], and Hopfield neural networks [12,13]. When the coexisting attractors reach an infinite number, this phenomenon is defined as extreme multistability [14], which has been reported in many memristor-based chaotic systems [15,16]. Similar to the continuous chaotic systems, the discrete chaotic maps are taken as a class of important dynamical systems, which can also give rise to the phenomenon of multistability. In the past decade, the coexistence of double or multiple attractors has been found in the Hénon maps [17,18], the M-dimensional nonlinear hyperchaotic model [19] and the multistage DC/DC switching converter [20]. Recently, two types of simple 2D hyperchaotic maps with sine trigonometric nonlinearity and constant controllers were shown to generate initial-boosted infinite attractors along a phase line [21,22]. Thereafter, a simple two-dimensional Sine map was presented to obtain the initials-boosted infinitely many attractors along a phase plane [23]. These newly presented discrete maps only exhibit the coexisting attractors with different positions. However, the coexisting infinite attractors with different topologies and different positions in the discrete maps are rarely reported.

Can a low-dimensional discrete map produce coexisting infinite attractors with different topologies and different positions? This paper reports the finding of such coexisting infinite orbits in an area-preserving Lozi map by numerical simulations. A Lozi map is a piecewise-linear map that was first reported in [24]. Under some typical control parameter settings, the Lozi map displays a singular hyperbolic chaotic attractor [25,26]. However, when the specific parameter settings are selected, the concerned Lozi map becomes a specific two-dimensional area-preserving map, and it can generate initial values-related coexisting infinite orbits. As such, the finding of coexisting infinite orbits in the area-preserving Lozi map is greatly significant.

The rest of this paper is organized as follows. Section 2 briefly reviews the Lozi map and then focuses on the area-preserving Lozi map. Section 3 discloses the coexisting infinite orbits and displays the initial values-switched iterative sequences. Section 4 calculates the complexity of the coexisting infinite orbits. Section 5 develops a microcontroller-based hardware platform to acquire the coexisting sequences. Finally, the paper gives a summary in Section 6.

## 2. Area-Preserving Lozi Map

This section briefly reviews the Lozi map and then focuses on the area-preserving Lozi map, including the fixed point stability and quasi-periodic route to chaos.

### 2.1. The Classical Lozi Map

A Lozi map [24,25] is two-dimensional and piecewise-linear, and is achieved by substituting the quadratic nonlinearity in Hénon map [27] with an absolute value nonlinearity. Its mathematical model can be rewritten as

(1) $$\left\{ \begin{array}{l} x_{n+1}=1-a\left|x_{n}\right|+y_{n}\\ y_{n+1}=bx_{n} \end{array}\right.$$

where the control parameters *a*, *b* are two real constants (*b* ≠ 0).

**Figure 1:**
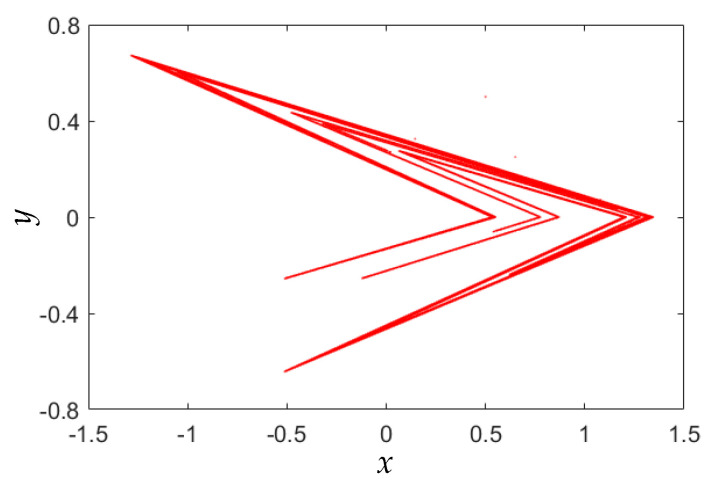
Chaotic attractor of Lozi map under typical parameter settings (*a* = 1.7 and *b* = 0.5).

Usually, the typical control parameters in the Lozi map are set as *a* = 1.7 and *b* = 0.5. Figure 1 gives a chaotic attractor generated from the Lozi map. By changing the control parameters, the Lozi map can display complex behaviors. This paper focuses on the finding of coexisting infinite orbits in the area-preserving Lozi map under the specific parameter settings.

### 2.2. Stability for the Fixed Points

The stability of the Lozi map model in (1) can be judged by its Jacobian matrix. The Jacobian matrix can be written as

(2) $$J=\left[\begin{array}{cc} -a\mathrm{sign}(x) & 1\\ b & 0 \end{array}\right]$$

Obviously, the determinant of ***J*** is −*b*, i.e., det(***J***) = −*b*. Particularly, when *b* = −1, the Lozi map is two-dimensional area-preserving [28].

Define the fixed point as ***F*** = (x*, y*) for the map model. The fixed point can be obtained by calculating the following equation set:

(3) $$\left\{ \begin{array}{l} x*=1-a\left|x*\right|+y*\\ y*=bx* \end{array}\right.$$

Due to the absolute value nonlinearity, Equation (3) is discussed in two cases, i.e., x* > 0 and x* < 0.

When x* > 0, the fixed point is expressed as ***F***_1_ = (*x*_1_*, *y*_1_*). If 1 + *a* – *b* > 0, the first fixed point ***F***_1_ is obtained by

(4) $$F_{1}=(x_{1}*,y_{1}*)=(\frac{1}{1+a-b},\frac{b}{1+a-b})$$

if not, the ***F***_1_ is nonexistent.

Analogously, when x* < 0, the fixed point is defined as ***F***_2_ = (*x*_2_*, *y*_2_*). If 1 − *a* – *b* < 0, the second fixed point ***F***_2_ is obtained by

(5) $$F_{2}=(x_{2}*,y_{2}*)=(\frac{1}{1-a-b},\frac{b}{1-a-b})$$

Otherwise, ***F***_2_ has no solution.

Substituting ***F***_1_ into (2), the Jacobian matrix at ***F***_1_ is rewritten as

(6) $$J=\left[\begin{array}{cc} -a & 1\\ b & 0 \end{array}\right]$$

The characteristic polynomial is yielded from (6) as

(7) $$P(\lambda )=\lambda^{2}+a\lambda -b$$

and the eigenvalues are obtained as

(8) $$\left\{ \begin{array}{l} \lambda_{1}=0.5(-a+\sqrt{a^{2}+4b})\\ \lambda_{2}=0.5(-a-\sqrt{a^{2}+4b}) \end{array}\right.$$

In the same way, the eigenvalues of the Jacobian matrix at ***F***_2_ are calculated as

(9) $$\left\{ \begin{array}{l} \lambda_{1}=0.5(a+\sqrt{a^{2}+4b})\\ \lambda_{2}=0.5(a-\sqrt{a^{2}+4b}) \end{array}\right.$$

**Figure 2:**
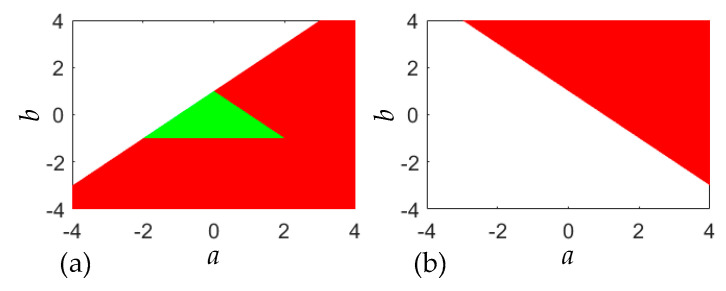
Stability distributions for the two fixed points in the control parameter plane of *a*, *b*. (**a**) stability distribution for the fixed point ***F***_1_; (**b**) stability distribution for the fixed point ***F***_2_.

If |*λ*_1,2_| < 1, the fixed point is stable; if |*λ*_1_| > 1 or |*λ*_2_| > 1, the fixed point is unstable. The eigenvalues *λ*_1,2_ are related to the two control parameters *a* and *b*. Thus, the stability distributions at the two fixed points depend on these control parameters. Because the fixed points ***F***_1,2_ are all in an unstable state without new change when |*a*| > 4 and |*b*| > 4, we denote that both *a* and *b* vary within the interval [−4, 4]. Figure 2 displays the stability distributions at the two fixed points in the control parameter plane of *a*, *b*, where the stable and unstable regions are marked in green and red, respectively. Meanwhile, Figure 2 also shows the variable regions of two control parameters.

From the aforementioned result, the Lozi map is two-dimensional area-preserving, as *b* = −1. For this particular case, the stability distributions at the two fixed points can be solved via (8) and (9). Based on (8), it can be found that when −2 ≤ *a* ≤ 2, the fixed point ***F***_1_ is in a critical state, i.e., |*λ*_1,2_| = 1; when *a* > 2, ***F***_1_ is unstable, i.e., |*λ*_1_| < 1 and |*λ*_2_| > 1. Similarly, it can be obtained from (9) that when *a* > 2, the fixed point ***F***_2_ is unstable, i.e., |*λ*_1_| > 1 and |*λ*_2_| < 1. The results indicate that when the control parameter *a* is in the interval [−2, 2], the Lozi map has only the fixed point ***F***_1_, and operates in a critical state.

### 2.3. Quasi-Periodic Route to Chaos

Using a bifurcation diagram, a finite-time Lyapunov exponent (LE) and a phase diagram, the parameter-dependent bifurcation behaviors and moving orbits can be analyzed. Note that the finite-time LE can be calculated by the Wolf’s Jacobian-based algorithm.

**Figure 3:**
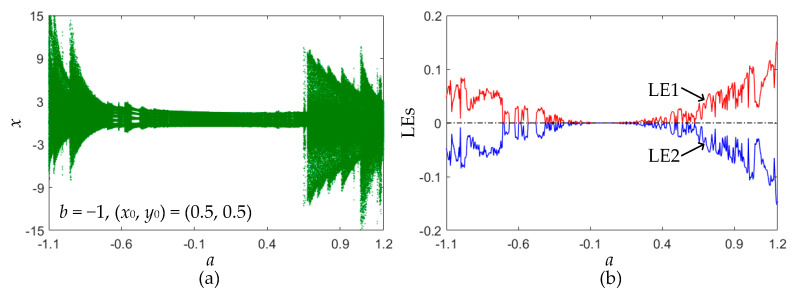
For fixed (*x*_0_, *y*_0_) = (0.5, 0.5), bifurcation plots of the area-preserving Lozi map with the increase in the control parameter *a*. (**a**) Bifurcation diagram; (**b**) finite-time LEs.

The particular case of the Lozi map with *b* = −1 is considered. For the area-preserving Lozi map, the initial values are determined as (*x*_0_, *y*_0_) = (0.5, 0.5), and the control parameter *a* varies in the interval [−1.1, 1.2]. This parameter variation interval implies that the Lozi map has only the critical stable fixed point ***F***_1_. The bifurcation diagram and finite-time LEs are plotted in Figure 3. As can be seen, with an increase in the control parameter *a*, the area-preserving Lozi map undergoes the quasi-periodic route to chaos. Therefore, the concerned Lozi map has a striking bifurcation route. Besides this, as observed from Figure 3b, LE_1_ is always non-negative and the sum of LEs equals zero, indicating the appearance of chaos and the conservation of the Lozi map [29].

**Figure 4:**
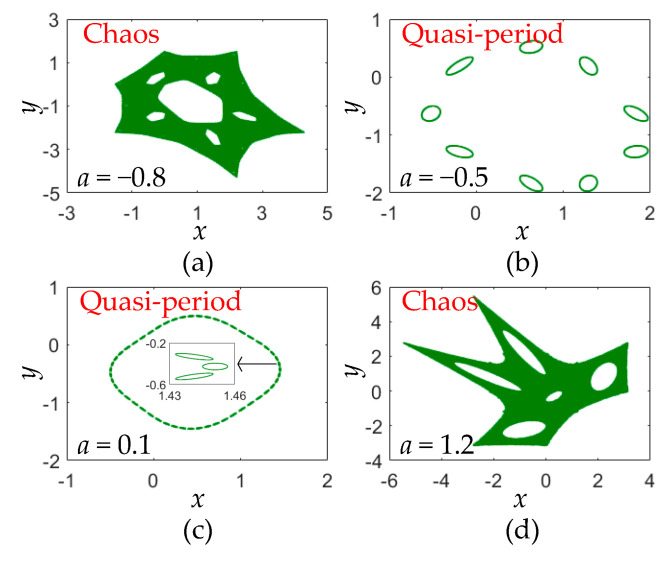
Phase orbits with different parameter *a.* (**a**) Chaos at *a* = −0.8; (**b**) quasi-period at *a* = −0.5; (**c**) quasi-period at *a* = 0.1; (**d**) chaos at *a* = 1.2.

Following the results in Figure 3, four phase diagrams of the area-preserving Lozi map for different parameter are obtained and shown in Figure 4. Figure 4a,d show two chaotic orbits with different complex fractal structures, whereas Figure 4b,c display quasi-periodic orbits with 9 and 51 tori. Therefore, the area-preserving Lozi map can generate chaotic and quasi-periodic orbits, and these moving orbits are entirely different from the chaotic attractor given by Figure 1.

## 3. Initial Values-Related Coexisting Infinite Orbits

The coexistence of infinite attractors is also called the phenomenon of extreme multistability, which has been reported in numerous memristor-based dynamical circuits and systems [14,15,16]. Mostly this phenomenon occurs in continuous dynamical systems of at least four dimensions. Hence the finding of coexisting infinite orbits in the area-preserving Lozi map is greatly significant.

### 3.1. Coexisting Chaotic and Quasi-Periodic Orbits

To explore the properties of the area-preserving Lozi map, we inspect its initial values-related dynamical behaviors via the dynamical map, the bifurcation diagram and the phase diagram. The control parameter *a* is fixed as 1.2. Note that the control parameter of *b* = −1 is kept unchanged to make sure the Lozi map is area-preserving.

**Figure 5:**
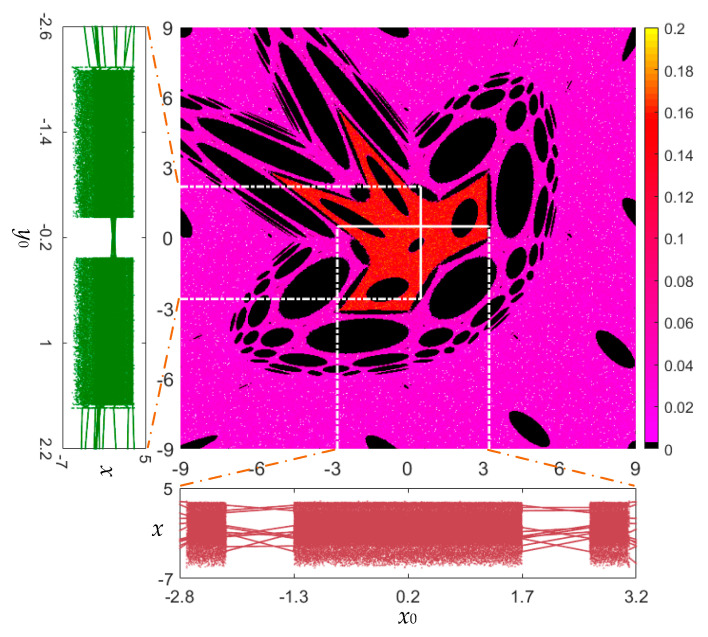
For fixed *a* = 1.2, *b* = −1, the colorful kinetic map (middle) determined by computing the values of LLE with respect to (*x*_0_, *y*_0_) ∈ [−9, 9] × [−9, 9]; local bifurcation diagram (left) for fixed *x*_0_ = 0.5 and variable *y*_0_ ∈ [−2.6, 2.2]; and local bifurcation diagram (bottom) for fixed *y*_0_ = 0.5 and variable *x*_0_ ∈ [−2.8, 3.2].

The largest LE (LLE) is a valuable indicator of chaos, and a colorful kinetic map can be plotted in the initial value plane by computing the values of LLE of a discrete map. Figure 5 (middle) displays the kinetic map of the area-preserving Lozi map with the initial value ranges of *x*_0_ ∈ [−9, 9] and *y*_0_ ∈ [−9, 9]. The initial value ranges emerging from the moving orbits with different values of LLE are labeled by different colors. The magenta–red–yellow, labeled as positive, represents the chaos areas and the dark, labeled as zero, represents the quasi-period and period areas. As can be observed, the kinetic map possesses a complicated fractal structure and smooth boundaries.

In terms of determining an initial value and taking another initial value as a bifurcation parameter, the initial value-related bifurcation diagrams of state *x* with respect to two initial values *x*_0_ and *y*_0_ are displayed in Figure 5 (left and bottom). Figure 5 (left) shows the local bifurcation diagram for fixed *x*_0_ = 0.5 and variable *y*_0_ ∈ [−2.6, 2.2], whereas Figure 5 (bottom) shows the local bifurcation diagram for fixed *y*_0_ = 0.5 and variable *x*_0_ ∈ [−2.8, 3.2]. The bifurcation diagrams intuitively manifest the dynamical transition from the quasi-periodic route to chaos as the initial values *x*_0_ and *y*_0_ individually change, resulting in the coexistence of infinite orbits.

**Figure 6:**
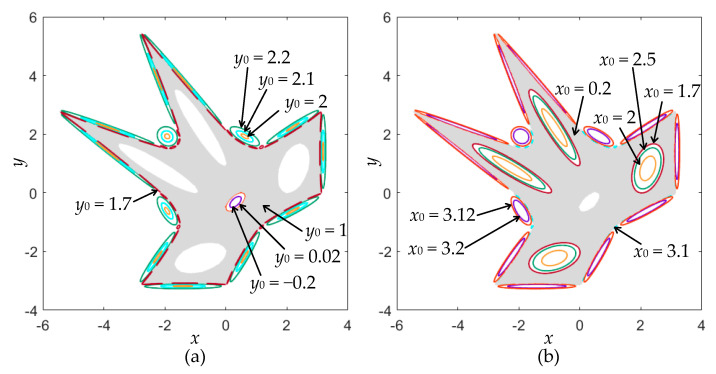
Coexistence of a chaotic orbit and vast quasi-periodic orbits. (**a**) Seven kinds of moving orbits under different *y*_0_ with *x*_0_ = 0.5; (**b**) seven kinds of moving orbits under different *x*_0_ with *y*_0_ = 0.5.

The phase diagram can reflect the moving orbits with different topologies, which is suitable to reveal the coexisting behaviors. With the bifurcation diagram given in Figure 5 (left), the phase diagrams under several different initial values *y*_0_ are depicted in Figure 6a, from which seven different chaotic and quasi-periodic orbits can be observed. In the same way, with the bifurcation diagram given in Figure 5 (bottom), the phase diagrams under several different initial values *x*_0_ are depicted in Figure 6b, from which seven different chaotic and quasi-periodic orbits can also be observed. Note that the chaotic orbit behaves like a chaotic sea, distributed in a determined region, and the quasi-periodic orbit appears as a closed torus. Therefore, the coexistence of a chaotic orbit with a fractal pattern and vast quasi-periodic orbits with different topologies can be disclosed in the area-preserving Lozi map.

### 3.2. Coexisting Chaotic and Periodic Orbits

**Figure 7:**
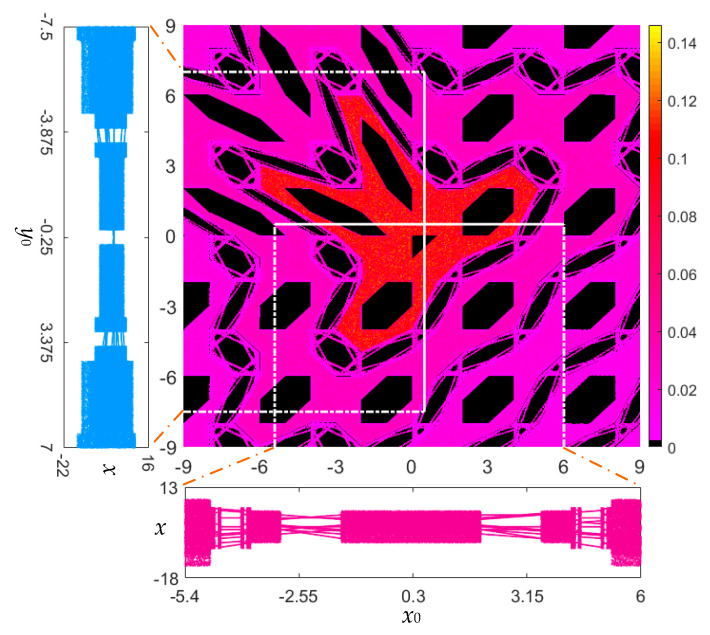
For fixed *a* = 1, *b* = −1, the colorful kinetic map (middle) determined by computing the values of LLE with respect to (*x*_0_, *y*_0_) ∈ [−9, 9] × [−9, 9]; local bifurcation diagram (left) for fixed *x*_0_ = 0.5 and variable *y*_0_ ∈ [−7.5, 7]; and local bifurcation diagram (bottom) for fixed *y*_0_ = 0.5 and variable *x*_0_ ∈ [−5.4, 6].

To further demonstrate the coexisting infinite orbits, the control parameter is set as *a* = 1 for the area-preserving Lozi map. Similar to the results in Figure 5, Figure 7 (middle) plots the kinetic map of the area-preserving Lozi map with the initial value ranges of *x*_0_ ∈ [−9, 9] and *y*_0_ ∈ [−9, 9]. The colorful areas in the initial value planes denote the same moving orbits as those used in Figure 5. As can be seen, the kinetic map in Figure 7 (middle) has a fractal structure different from that of Figure 5.

For this case, when fixing an initial value and taking another initial value as a bifurcation parameter, the initial value-related bifurcation diagrams of state *x* with respect to two initial values *x*_0_ and *y*_0_ are displayed in Figure 7 (left and bottom). Figure 7 (left) shows the local bifurcation diagram for fixed *x*_0_ = 0.5 and variable *y*_0_ ∈ [−7.5, 7], whereas Figure 7 (bottom) shows the local bifurcation diagram for fixed *y*_0_ = 0.5 and variable *x*_0_ ∈ [−5.4, 6]. Different from the bifurcation diagrams in Figure 5 (left and bottom), the bifurcation diagrams embody the dynamical transition from the periodic route to chaos as the initial values *x*_0_ and *y*_0_ individually change.

**Figure 8:**
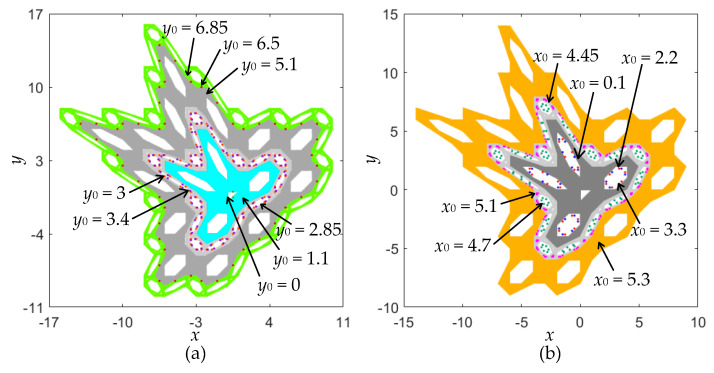
Coexistence of chaotic and periodic orbits. (**a**) Eight kinds of coexisting orbits under different *y*_0_ with *x*_0_ = 0.5; (**b**) seven kinds of coexisting orbits under different *x*_0_ with *y*_0_ = 0.5.

Based on the bifurcation diagrams given in Figure 7, the phase diagrams initiated by different initial values are shown in Figure 8. Figure 8a gives eight kinds of coexisting orbits under a different initial value *y*_0_ with *x*_0_ = 0.5, and Figure 8b shows seven kinds of coexisting orbits under a different initial value *x*_0_ with *y*_0_ = 0.5. Therefore, the coexistence of chaotic and periodic orbits with different types and topologies can be disclosed in the area-preserving Lozi map as well. It is stressed that the periodic orbit given in Figure 8 only manifests as some discrete points, whereas the quasi-periodic orbit appears as a closed torus, shown in Figure 6.

Consequently, when the Lozi map is area-preserving, its moving orbits are extremely dependent on its initial values and present period, quasi-period and chaos, with different types and topologies. In other words, the emergence of extreme multistability appears in the area-preserving Lozi map.

### 3.3. Initial Values-Switched Iterative Sequences

**Figure 9:**
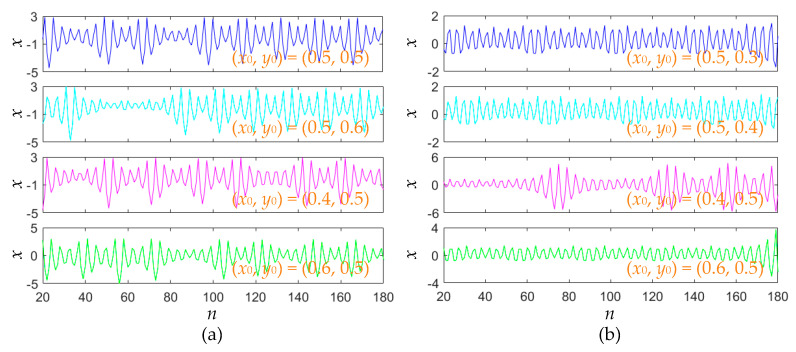
Initial values-related chaotic iterative sequences. (**a**) Chaotic sequences for *a* = 1.2; (**b**) chaotic sequences for *a* = 1.

Corresponding to the results in Figure 6 and Figure 8, the initial value-related iterative sequences can be obtained from the area-preserving Lozi map. For fixed *a* = 1.2, four sets of initial values, (0.5, 0.5), (0.5, 0.6), (0.4, 0.5) and (0.6, 0.5), are selected. Meanwhile, for determined *a* = 1, another four sets of initial values, (0.5, 0.3), (0.5, 0.4), (0.4, 0.5) and (0.6, 0.5), are chosen. For these sets of initial values, all the iterative sequences are chaotic, and they can be depicted in Figure 9 by numerical simulations.

**Figure 10:**
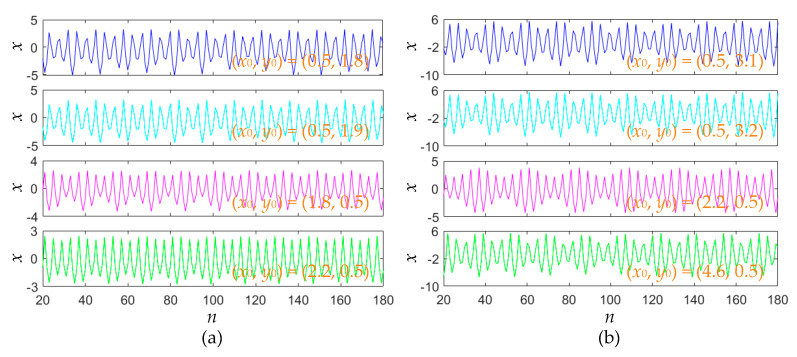
Initial values-related quasi-periodic and periodic iterative sequences. (**a**) Quasi-periodic sequences for *a* = 1.2; (**b**) periodic sequences for *a* = 1.

Except for the chaotic sequences, the area-preserving Lozi map can also produce quasi-periodic and periodic sequences. For fixed *a* = 1.2, the initial values are set to (0.5, 1.8), (0.5, 1.9), (1.8, 0.5), and (2.2, 0.5), respectively. In these cases, four sets of quasi-periodic sequences are generated and shown in Figure 10a. Thereafter, for fixed *a* = 1, the initial values are assigned as (0.5, 3.1), (0.5, 3.2), (2.2, 0.5), and (4.6, 0.5), respectively. In these cases, four sets of periodic sequences are generated and shown in Figure 10b.

## 4. Initial Values-Related Complexity

The spectral entropy(SE) and sample entropy (SampEn) are two useful measures of chaos, which can be used to quantitatively calculate the complexity of the coexisting infinite orbits.

### 4.1. SE-Based Complexity

The SE indicates the disorder of time sequences using an algorithm to measure complexity based on the Fourier transformation [30]. The higher the SE is, the higher the complexity of the time sequences will be.

For a set of time sequences {*x_n_*, *n* = 0, 1, 2, …, *N*−1} with a length of *N*, a new discrete number of length *N* is obtained by subtracting the mean of this dataset from each datum, which is described as

(10) $$x_{n}=x_{n}-\frac{\sum_{n=0}^{N-1}x_{n}}{N}$$

Denote *X_k_* as the discrete Fourier transform of *x_n_*. This yields

(11) $$X_{k}=\sum_{n=0}^{N-1}x_{n}e^{-j2\pi nk/N}$$

in which *k* = 0, 1, 2, …, *N* − 1 and j is the unit imaginary. The probability of the power spectrum is defined by

(12) $$P_{k}=\frac{{\left|X_{k}\right|}^{2}}{\sum_{k=0}^{N/2-1}{\left|X_{k}\right|}^{2}}$$

Therefore, the normalization spectral entropy can be obtained as

(13) $$\mathrm{SE}=\frac{\sum_{k=0}^{N/2-1}\left|P_{k}\ln(P_{k})\right|}{\ln(N/2)}$$

where *P_k_* = 0 needs to be removed, or let *P_k_*ln*P_k_* = 0 when *P_k_* = 0. According to the definition of SE, the SE-based complexity can be obtained when the time sequences of state *x* are selected for the area-preserving Lozi map.

**Figure 11:**
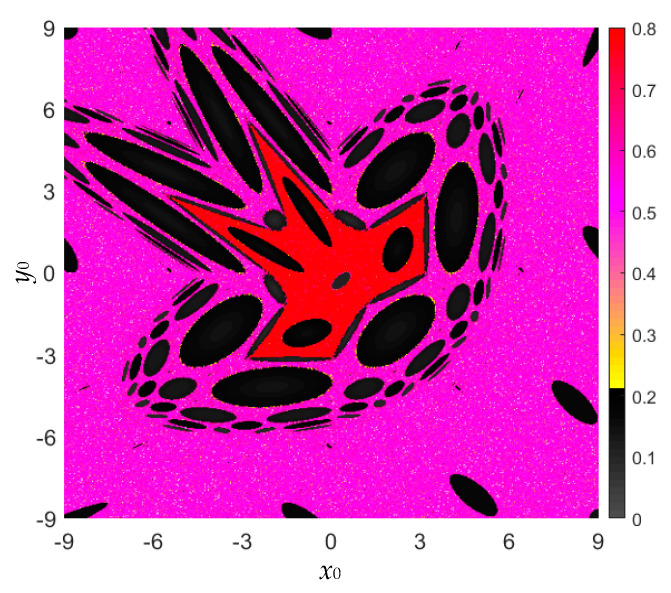
For fixed *a* = 1.2, *b* = −1, the colorful SE-based complexity distribution with respect to (*x*_0_, *y*_0_) ∈ [−9, 9] × [−9, 9].

Following the kinetic map in Figure 5, the initial value ranges for the area-preserving Lozi map are also considered as *x*_0_ ∈ [−9, 9] and *y*_0_ ∈ [−9, 9]. The SE-based complexity distributed in the initial value plane is shown in Figure 11. It is not difficult to find that the SE-based complexity distribution is consistent with the kinetic map in Figure 5 (middle).

### 4.2. SampEn-Based Complexity

The sample entropy (SampEn) is proposed by reference to the approximate entropy and can reflect the regularity of time sequences [31]. When evaluating sequence complexity, a larger SampEn indicates lower regularity, i.e., greater complexity.

In the same way, consider a set of time sequences {*x_n_*, *n* = 1, 2, 3, …, *N*} with a length of *N*. This set of time sequences constructs the (*N* − *m* + 1) *m*-dimensional vectors ***X****_m_*(*i*), where ***X****_m_*(*i*) = {*x_i_*, *x_i_*_+1_, …, *x_i_*_+*m*–1_}, 1 ≤ *i* ≤ *N* − *m* + 1. The distance between two vectors ***X****_m_*(*i*) and ***X****_m_*(*j*) is defined as the absolute value of the maximum difference in the corresponding element, i.e., *d*[***X****_m_*(*i*), ***X****_m_*(*j*)] = max{|*x*(*i* + *k*) − *x*(*j* + *k*)|}, where 0 ≤ *k* ≤ *m* − 1. The ***B****_i_* is the number of *d*[***X****_m_*(*i*), ***X****_m_*(*j*)] < *r* (1 ≤ *j* ≤ *N* – *m*, *i* ≠ *j*). Define (*N* − *m* + 1)^−1^ times ***B****_i_* as

(14) $$B_{i}^{m}(r)=\frac{1}{N-m-1}B_{i}$$

and define the probability ***B***^(*m*)^(*r*) that two sequences will match for *m* points as

(15) $$B^{\left(m\right)}(r)=\frac{1}{N-m}\sum_{i=1}^{N-m}B_{i}^{m}(r)$$

Similarly, ***X****_m_*_+1_(*i*) is (*m* +1)-dimensional vectors and ***A****_i_* is the number of *d*[***X****_m_*_+1_(*i*), ***X****_m_*_+1_(*j*)] < *r.* The (*N* − *m* + 1)^−1^ times ***A****_i_* and the probability, ***A***^(*m*)^(*r*), that two sequences will match for *m* +1 points are

(16) $$A_{i}^{m}(r)=\frac{1}{N-m-1}A_{i}$$

(17) $$A^{\left(m\right)}(r)=\frac{1}{N-m}\sum_{i=1}^{N-m}A_{i}^{m}(r)$$

Then, when *N* is finite, the sample entropy can be calculated by

(18) $$\mathrm{SampEn}=-\ln\left[\frac{A^{m}(r)}{B^{m}(r)}\right]$$

where *m* = 2 and *r* = 0.2 times the standard deviation in our experiment based on previous studies [32].

**Figure 12:**
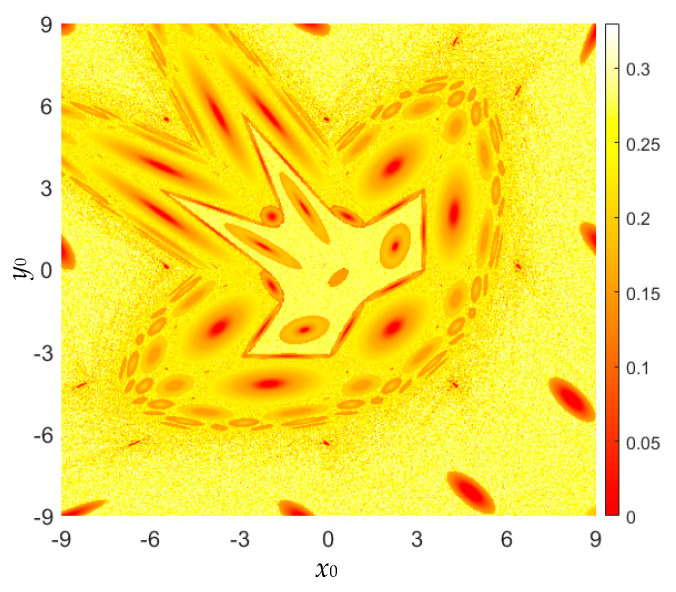
For fixed *a* = 1.2, *b* = −1, the colorful SampEn-based complexity distribution with respect to (*x*_0_, *y*_0_) ∈ [−9, 9] × [−9, 9].

The time sequences of state *x* generated by the area-preserving Lozi map are presumed to be the measurable sequences. According to the kinetic map in Figure 5, the initial value ranges are also considered as *x*_0_ ∈ [−9, 9] and *y*_0_ ∈ [−9, 9]. The SampEn-based complexity distributed in the initial value plane is plotted in Figure 12. Comparing the results in Figure 11 and Figure 12, the SE-based complexity and SampEn-based complexity have the same distribution in the initial value plane.

## 5. Hardware Experiment

To physically implement the simple area-preserving Lozi map, a digital microcontroller-based hardware platform is developed. It mainly includes the microcontroller STM32F407VET6 (ARM 32-bit Cortex™-M4 CPU), a D/A converter TLV5618 (12-bit) and a unipolar/bipolar conversion circuit consisting of operational amplifier and resistor. The STM32F4 series chip with flexible pin configuration is easy to weld, which allows us to design a feature-rich printed circuit board (PCB) by matching different peripheral circuits for multiple engineering applications. Of course, in order to achieve more complex chaos-based applications, the raspberry pi board with superior performance can be used to complete the physical implementation by referring to [33].

**Figure 13:**
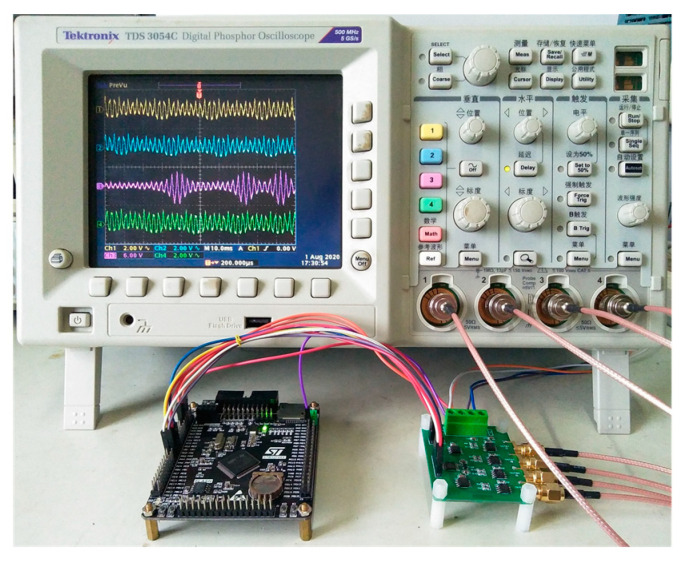
Digital microcontroller-based experiment platform and the displayed four-channel waveforms.

The model equations, control parameters and initial values are written using C language, and the program is downloaded to the microcontroller. For convenience, Algorithm 1 lists the pseudocode of the microcontroller-based main program [34]. Then, the time-domain waveforms generated by the hardware platform can be clearly observed in the oscilloscope. The screenshot of the four-channel waveforms obtained by the oscilloscope and experimental hardware are shown in Figure 13.
**Algorithm 1** The microcontroller-based main programInitialize the microcontroller and configure the output pins; Set the length *N* of sequences, the Number *M* of interpolation; Set intermediate variables *step*_1,2,3,4_, *temp*_1,2,3,4_ and *value*_1,2,3,4_; **while** true Set four sets of (*a_i_*, *b_i_*, *x_i_*_,0_, *y_i_*_,0_) (*i* = 1,2,3,4); **for**
*i* = 1 to *N*   //system equation   *x*_1,*i*+1_ = 1 − *a*_1_|*x*_1,*i*_| + *y*_1,*i*_; *y*_1,*i*+1_ = *b*_1×1,*i*_;   …   *x*_4,*i*+1_ = 1 − *a*_4_|*x*_4,*i*_| + *y*_4,*i*_; *y*_4,*i*+1_ = *b*_4×4,*i*_;   //interpolation   *step*_1_ = (*x*_1,*i*+1_ − *x*_1,*i*_)/*M*; … *step*_4_ = (*x*_4,*i*+1_ − *x*_4,*i*_)/*M*;   **for**
*j* = 0 to *M* − 1    *temp*_1_ = *x*_1,*i*_ + *j*∙*step*_1_; *value*_1_ = (*temp*_1_ + 15)*4096/30;    …    *temp*_4_ = *x*_4,*i*_ + *j*∙*step*_4_; *value*_4_ = (*temp*_4_ + 15)*4096/30;    Transfer the *value*_1,2,3,4_ to TLV5618;   **end** **end**

**Figure 14:**
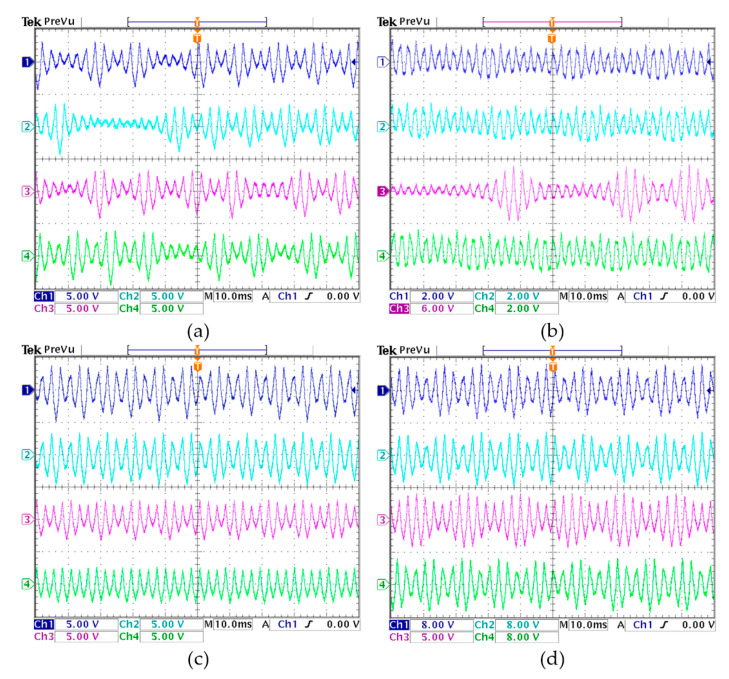
Initial values-related time-domain waveforms generated from the hardware platform. (**a**) Chaotic waveforms with *a* = 1.2; (**b**) chaotic waveforms for *a* = 1; (**c**) quasi-periodic waveforms for *a* = 1.2; (**d**) periodic waveforms for *a* = 1.

With the digital hardware platform, the discrete iterative sequences for two concerned cases are measured experimentally. Corresponding to the numerical results in Figure 9 and Figure 10, the initial values-related time-domain waveforms generated from the hardware platform are displayed in Figure 14. The experimental results demonstrate that the chaotic, quasi-periodic and periodic waveforms for fixed *a* = 1 and 1.2 can be captured by the oscilloscope. Because the sequence sampling is random, the initial values-related time-domain waveforms obtained in the experiments are slightly different from the numerically simulated time-domain waveforms. Ignoring these tiny errors, the experimental results are consistent with the numerical results.

## 6. Conclusions

In order to explore the initial values-related coexisting infinite orbits in a discrete dynamical system, this paper studied the two-dimensional area-preserving Lozi map under specific parameter settings. Within the control parameter of interest, the area-preserving Lozi map has only a fixed point and operates in a critical state. Since the critical fixed point is extremely sensitive to the initial values, the area-preserving Lozi map can easily generate the initial values-related coexisting infinite orbits, i.e., extreme multistability, including periodic, quasi-periodic and chaotic orbits with different types and topologies. The SE- and SampEn-based complexity distributions were employed to evaluate the dynamical performance of the sequences generated from different initial values. By switching the initial values, the iterative sequences were acquired from the developed hardware experiment platform. Of course, the initial values-switched chaotic iterative sequences are worthy of further study in some practical applications.
